# MALIGNANT PLEURAL MESOTHELIOMA WITHOUT ASBESTOS EXPOSURE WITH DISTANT METASTASIS IN A PERIPHERAL LYMPH NODE: A CASE REPORT

**DOI:** 10.4103/0970-2113.44137

**Published:** 2008

**Authors:** Surya Kant, Sanjay Kumar Verma, Sanjay

**Affiliations:** Department of Pulmonary Medicine

**Keywords:** Malignant mesothelioma, asbestos, lymph nodes

## Abstract

**SUMMARY:**

Malignant mesothelioma is an uncommon pleural neoplasm and usually associated with inhalation exposure to asbestos. About 20% of the patients have no demonstrable exposure to asbestos. It rarely metastasizes in peripheral lymph nodes. Here is a case report of malignant pleural mesothelioma without asbestos exposure with cervical lymph node metastasis

## INTRODUCTION

The most common primary malignant tumor of the pleura is malignant mesothelioma, an insidious neoplasm with a dismal prognosis arising from the mesothelial surfaces of the pleura. Malignant mesothelioma may metastasize to lymph nodes outside thorax.^1^ However, distant metastases are rare, and it is exceptional for patients to present with lymphadenopathy prior to the discovery of the primary tumor.^2^

## CASE REPORT

A 68 years male, retired clerk and chronic bidi smoker was admitted to our department with complaint of right sided pleuritic chest pain for 1 month and a painful right cervical mass. No occupational or domestic contact with asbestos could be elicited. Physical examination revealed a single, hard, moveable and tender right sided cervical lymph node of size 4x4 cm. Respiratory system examination revealed stony dull note and absent breath sounds in right infraxillary and infrascapular regions. Examination of other systems was unremarkable.

**Fig 1:**
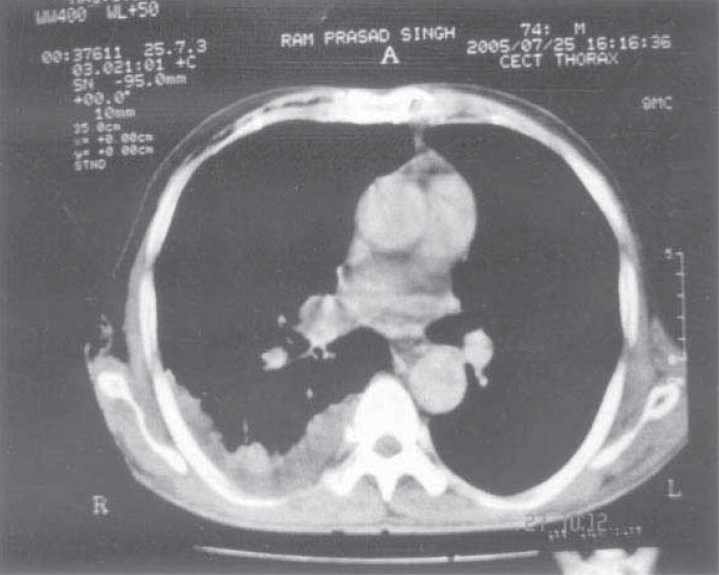
CT thorax showing right sided pleural effusion.

His hematological and bio-chemical investigations were within normal limits. His chest x-ray PA view revealed right sided pleural effusion. His PPD was negative. His sputum smear for AFB and malignant cells was negative. His CT-Thorax revealed right sided mediastinal lymphadenopathy (right Para tracheal and precarinal) with right sided pleural effusion (fig. 1).

**Fig 2:**
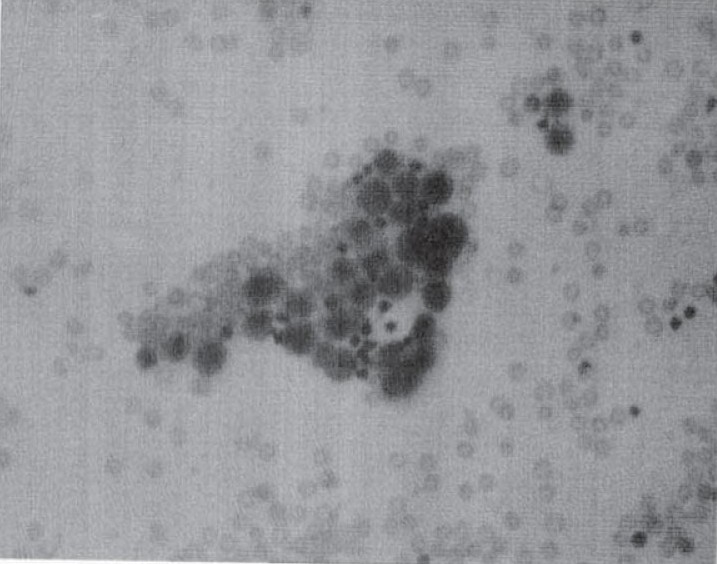
Pleural fluid showing malignant mesothelial cells

Pleural fluid aspiration was hemorrhagic in colour. Pleural fluid examination revealed: Protein: 3.36 gm%, sugar: 75mg%, Total cell count: 4000/mm^ 3^, Differential Leukocyte Count: L81, P19, Lactic Dehydrogenase: 3775 U/ml, Adenosine Deaminase: 23.60 U/L along with large numbers of mesothelial cells. His pleural fluid cytology revealed malignant mesothelioma (fig. 2). Closed pleural biopsy was done which was nondiagnostic. Open pleural biopsy was planned but patient did not give consent for this.

**Fig 3 & 4:**
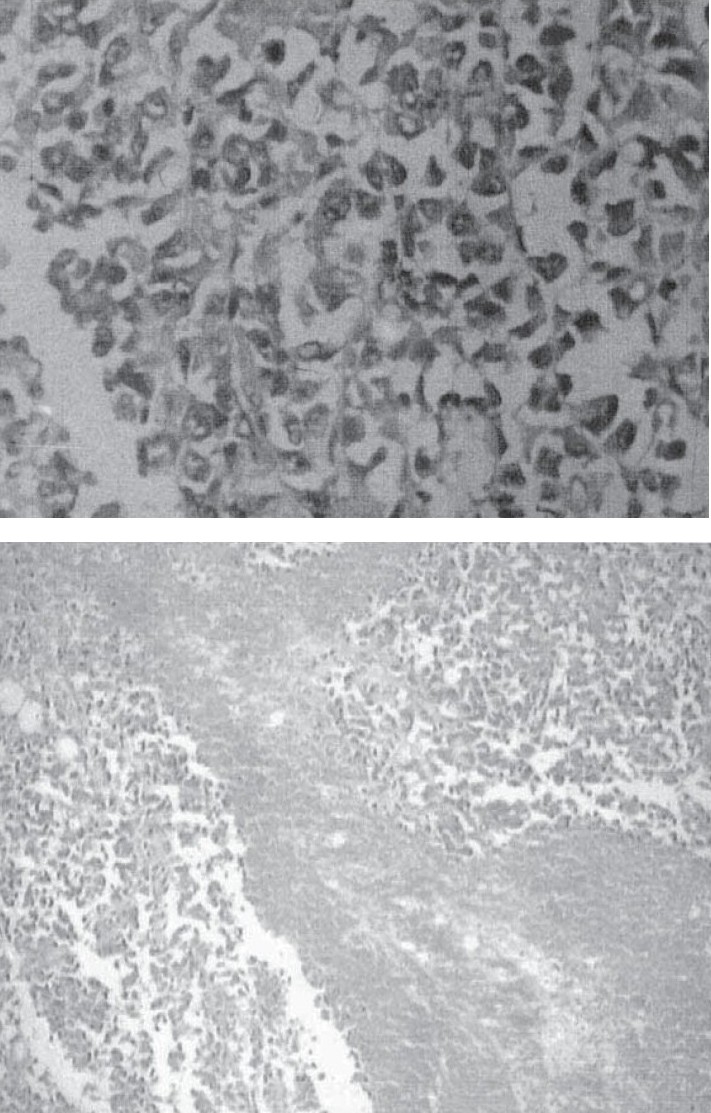
Histopathology of lymph node showing atypical mesothelial cells infiltrating the lymph node.

Biopsy of right sided cervical lymph node revealed atypical proliferated cuboidal or columnar epithelial cells arranged in an irregular tubo-papillary pattern with fair degree of anisonucleosis and angulation of cell outline (fig. 3 and 4). Immunohistochemical stains showed positivity for Pancytokeratin and negative reactivity for CEA and CD-15 (Leu-M_ 1_). Thus the diagnosis of malignant mesothelioma was confirmed.

The case was considered as M1 in stage IV according to the International Mesothelioma Interest Group (IMIG) staging system^3^, and in stage III according to the Butchard staging system^4^. His treatment was planned but patient expired just after 2 days of hospital stay from respiratory failure.

## DISCUSSION

Pleural mesothelioma typically presents with chest pain and pleural effusion^5^, as in this case. The effusions are frequently large, occupying 50% or more of the hemithorax and obscuring the pleural tumour. The tumour generally spreads by local invasion throughout the pleural cavity to the chest wall and to the axilla and supraclavicular area. The diaphragm, as well as the surface of the peritoneum or intraabdominal organs, may also be involved.

Distant metastatic disease is unusual^6^ in mesothelioma but may involve the liver, bone, brain, adrenal gland, kidney, pancreas, thyroid, spleen, skin and lymph nodes. Hilar and mediastinal lymph node involvement occurs in less than 50% of patients. Extrathoracic lymph node involvement is very rare. Cervical lymph node metastasis has been reported at autopsy^7^. Clinically documented distant lymph node metastasis from pleural mesothelioma is very rare^8^.

The mean age of patients with malignant mesothelioma is approximately 60 years^9^ (in our case the age was 60 years) but the disease can occur at any age, including childhood. The male to female ratio is 4: 1^10^ with a predominance of right side over the left (60: 40)^11^.

Mesothelioma has a strong relationship with asbestos exposure. About 20% of the patients have no demonstrable exposure to asbestos^12^–^13^. Possibility of association of malignant mesothelioma and exposure to some dust or chemicals (stone cutter, leather factory or textile factory worker, agricultural chemicals) has also been reported. In the present case, the patient was a retired clerk and was not exposed to any of the known etiological factors. Our patient was a chronic smoker and it can be presumed that smoking could possibility have led to malignant mesothelioma of the pleura.

Histologically malignant mesotheliomas are of three types: Epithelial, Sarcomatoid and Mixed. Epithelial type is commonest which is seen in 50 to 60 percent of cases. Immunohistochemical assays developed for specific antigens are often helpful in differentiating mesothelioma from metastatic adenocarcinoma of various origins. Such antigens include carcinoembryonic antigen, CD15 (Leu-M1), and epithelial membrane antigen. Carcinoembryonic antigen and CD15 are typically absent in Malignant Pleural Mesothelioma^14^.

Diagnosis of malignant mesothelioma by non- invasive techniques like chest radiography, bronchoscopy, pleural fluid cytology and CT scan is rarely confirmatory^15^. In malignant mesothelioma pleural fluid is usually exudative with lymphocytic predominance (71 % was in present case). Pleural fluid LDH is usually more than 600 IU/ml; while in our case it was 3775 IU/ml. Open pleural biopsy of the tumour mass is most often required for confirmation of diagnosis^16^.

Many anticancer agents have been studied in patients with MPM, either as single agents or as part of a combination chemotherapy regimen. Response rates have ranged from 0 to 48%, with the highest response rates generally achieved using multiagent regimens. However, no clear standard of care using available agents has emerged due to a lack of clearly demonstrated survival or palliative benefit in the setting of well-designed, randomized, and controlled clinical trials. Radiation has shown palliative benefit in reducing pain and symptoms of dyspnea. Surgical pleurodesis can reduce the symptoms associated with recurrent or persistent pleural effusions, and chemotherapy has demonstrated palliative benefit in terms of overall quality of life^17^.

In conclusion, although extrathoracic lymph node metastases in malignant mesothelioma are very rare, nevertheless, in suspected cases, lymph node biopsy needs to be performed for the diagnosis of the metastatic disease.
